# Incidence of primary bone sarcomas in Iranian population (2008-2015): A national population-based study

**DOI:** 10.22088/cjim.13.4.741

**Published:** 2022

**Authors:** Adel Ebrahimpour, Mehrdad Sadighi, Amin Karimi, Amir Sabaghzadeh, Farsad Biglari, Mohammadreza Chehrassan, Mehdi Azizmohammad Looha, Meisam Jafari Kafiabadi, Mohammad Esmaeil Akbari, Amin Nokhostin-Ansari

**Affiliations:** 1 Cancer Research Center, Shahid Beheshti University of Medical Sciences, Tehran, Iran; 2 Department of Orthopedic Surgery, Shohadaye Tajrish Hospital, Shahid Beheshti University of Medical Sciences, Tehran, Iran; 3 Department of Orthopedic Surgery, Taleghani Hospital, Shahid Beheshti University of Medical Sciences, Tehran, Iran; 4 Department of Biostatics, Faculty of Paramedical Sciences, Shahid Beheshti University of Medical Sciences, Tehran, Iran; 5 Sports Medicine Research Center, Neuroscience Institute, Tehran University of Medical Sciences, Tehran, Iran

**Keywords:** Osteosarcoma, Neoplasms, Bone tissue, Epidemiology, Incidence

## Abstract

**Background:**

Epidemiological characteristics of bone sarcomas are variant in different populations, however, there is no previous study on primary bone sarcomas among Iranian population. This study aimed to evaluate the incidence, age, sex distribution, histologic type, and location of malignant bone sarcomas, based on the Iran National Cancer Registry (INCR).

**Methods:**

This was a national population-based study using INCR data from March 20, 2008, to March 20, 2015, on patients who were diagnosed with primary bone sarcomas of the appendicular (C-code:40) and axial skeleton (C-code 41), excluding skull and face bones. Primary bone sarcomas were classified according to the International Classification of Diseases for Oncology (ICD-O-3: C40–C41).

**Results:**

A total of 4112 patients (59.5% males and 40.5% females) with a mean age of 36 years were included in the study. 60.38% of patients were between 10 to 44 years old. The overall age-standardized incidence rates (ASIR) was 8.23 (males=9.67 and females=6.80) per million person-years. Osteosarcoma chondrosarcoma and Ewing sarcoma were the three main histology subtypes with the ASIR of 2.36, 1.26, and 1.08 per million person-years. Long bones of the lower limb were the most affected area, with the ASIR of 3.18 (95% CI: 3.02-3.33) per million. We found an increasing trend in the incidence of bone sarcomas in Iran from 8.59 in 2007 to 11.37 per million person-year in 2015.

**Conclusion:**

This study provided the epidemiological features of bone sarcomas, including the histological type of sarcoma, tumors’ location, and patients’ age and gender in the Iranian population for the first time.

Sarcomas are heterogeneous tumors, consisting of about fifty tumors that arise from mesenchymal cells (1). Sarcomas are categorized into two major groups, soft tissue and bone sarcomas (2). Only10% of sarcomas arise from bone, and the rest originate from soft tissue (3). Chondrogenic, osteogenic, fibrogenic, osteoclastic giant cell-rich, notochordal, and vascular tumors, Ewing sarcoma, and undifferentiated high-grade pleomorphic sarcoma are categorized as types of bone sarcomas (2). Bone sarcomas are relatively rare, with a prevalence of 0.2% in populations (4). Osteosarcoma is the most prevalent primary bone sarcoma, followed by chondrosarcoma and Ewing sarcoma (5). Bone sarcomas are different from each other in terms of epidemiological distribution. Osteosarcoma and Ewing sarcoma are more common in people in the second decade of their lives, but chondrosarcoma is common in the fifth and sixth decades (5). During recent decades, progression in the early detection and treatment of cancer have led to improvements in survival rate (6).

Although primary bone sarcomas are rare, they are a major cause of mortality and morbidity among patients with cancer, with 5- year relative survival rates of 53–55% (7). The geographic distribution of bone sarcoma worldwide appears to be quite variable, with a very low incidence reported in some Asian countries and Latin America. There seems to be an increased incidence among patients of African American and Caribbean descent over whites (8, 9).

There are several studies regarding the epidemiology of sarcomas in Iran. Sadighi et al. investigated the epidemiology of sarcomas in Iran. In their study, the mean age of patients with sarcomas in Iran was 30 years, and the male to female ratio was 1.6:1. Osteosarcoma was the most common bone sarcoma in adults and pediatric populations (10). In another study, Solooki et al. evaluated the epidemiology of musculoskeletal tumors in Iran. They found osteosarcoma as the most common malignant bone tumor accounting for 50.6% of malignant bone tumors (11). Ebrahimpour et al. reported the epidemiology of Ewing sarcoma in Iran. The mean age of patients was 21.53 years, and it was more common in males than females (12). 

Although there are studies on the epidemiology of sarcomas there is no study evaluating bone sarcomas’ overall epidemiology and comparing epidemiological features of different bone sarcoma types (13). This study’s objective was to review the incidence, age and sex distribution, histologic type, and location of all malignant bone tumors based on data derived from the Iran National Cancer Registry (INCR) (14, 15). Increasing knowledge of the incidence, prevalence, and distribution patterns of bone sarcomas will help orthopedic surgeon provide better health cares to improve outcomes for patients who suffer from bone sarcomas.

## Methods

**Study population: **This was a population-based retrospective cohort study using INCR data from March 20, 2008, to March 20, 2015, on patients who were diagnosed with primary bone sarcomas. Registration was mandatory and all hospitals, laboratories, and clinics had to submit medical records information to the INCR All data were classified into three formats: pathological test, clinical records or death certificate in the INCR. Due to the time-consuming steps of data collection, preparation and organization, access to national data was only possible until 2015. 

**Data variables: **Data, including patients’ age, sex, tumor location, tumors’ histological features, and city of residence, were derived from the registry and recorded. Furthermore, primary bone sarcomas of the appendicular (C-code:40) and axial skeleton (C-code 41), excluding skull and face bones, were classified according to the third edition of the International Classification of Diseases for Oncology (ICD-O-3) (16). All histology subtypes of primary bone sarcomas including ‘osteosarcoma,’ ‘chondrosarcoma,’ ‘malignant giant cell tumor (GCT),’ ‘Ewing sarcoma,’’ plasmacytoma,’ ‘other specified sarcoma (including fibrosarcoma, synovial sarcoma, hemangiosarcoma, neurofibrosarcoma),’ ‘sarcoma, not otherwise specified (NOS: including undifferentiated sarcoma and malignant tumor, fusiform cell type),’ and ‘other malignancy were derived and registered. 

**Data Quality: **Data quality was evaluated first by INCR. Accordingly, in the first step, the compliance of the recorded morphology and topography codes for each patient was assessed. If the irrelevant morphology code was recorded, the data were re-checked and if the data was recorded incorrectly, that case was removed from the study. In the next step, the equality of the registered year of birth for each patient and her/his age was evaluated; any discrepancies between the year of birth and age caused the patients' records to be re-checked. In this study, only microscopically and pathologically confirmed cases of bone sarcoma were derived from data registry. Therefore, other data were dropped from dataset. 

Data cleaning such as removing wrong histology subtypes, topography codes and duplicate cases were conducted by authors. Patients with the same first name, surname, and father’s name were identified as duplicate records and excluded from the study.

**Statistical analysis: **Descriptive statistics were expressed by mean, median, Interquartile range (IQR) for numeric variable and frequency (percentage) for categorical variables. The age-specific incidence rates were reported for each age group across different topography codes as follows [1]: (17)

Number of new cases of diseasePopulation at risk in a period of time 

The age-standardized incidence rates (ASIRs) per million were calculated according to gender, year of diagnosis, and histology subtypes using the new World Health Organization (WHO) standard population and the direct standardization method [2]: (18)

$\mathrm{ASIR}=\frac{\sum_{i=1}^{A}a_{i}w_{i}}{\sum_{i=1}^{A}W_{i}}$

where w_i_ and a_i_ were the population weights and age specific rate for ith age class (i=1,2,…,A). The 95% confidence interval (95% CI) of ASIRs was expressed using the direct method [1]. All analyses were performed in R (version 3.6.0) and IBM SPSS (Version 26) software. A p-value lower than 0.05 was considered as statistically significant. 

## Results

A total of 4112 patients were included in the study, of whom 2445 (59.5%) were males, and 1667 (40.5%) were females. Males were significantly more affected by bone sarcomas compared to females (P<0.001). Osteosarcoma, chondrosarcoma, Ewing sarcoma, and plasmacytoma affected males more (pp<0.05). The mean age of participants at the time of diagnosis was 36 years, and there was no significant difference between males and females in this regard (P=0.915). Mean age of males with plasmacytoma was not otherwise specified (NOS) and Ewing sarcoma was higher compared to females (P<0.05), but there was no significant difference between males and females with other types of bone sarcomas regarding the age at the time of diagnosis (p>0.05). Results showed that osteosarcoma was the most common histological subtypes, followed by chondrosarcoma, Ewing sarcoma, plasmacytoma NOS, and giant cell tumor of bone (table 1). Furthermore, the osteosarcoma NOS and chondrosarcoma NOS were the most common subtypes of osteosarcoma and chondrosarcoma, respectively. Gender distribution of different types of bone sarcoma and mean age of patients at the time of diagnosis based on the histological type of the bone sarcomas were reported in table 1. 

**Table 1:** Incidence of primary bone sarcomas in Iran from 2008-2015 (n=4112)

**Histology Subtype**	**Frequency (Percentage)**				**Mean age at diagnosis (Median, range)**			
	**Total**	**Male**	**Female**	**Exact** P-value*	**Total**	**Male**	**Female**	**Exact** P-value**
Osteosarcoma	1409 (100)	864(61.3)	545(38.7)	<0.001	25 (20, 0-91)	25 (20, 0-87)	25 (19, 0-91)	0.199
Chondrosarcoma	611(100.0)	362 (59.2)	249 (40.8)	<0.001	45 (44, 0-93)	45 (44, 0-87)	45 (44, 0-93)	0.942
Ewing Sarcoma	602 (100.0)	373 (62.0)	229 (38.0)	<0.001	21 (19, 0-80)	22 (20, 0-80)	20 (18, 1-75)	0.004
Giant Cell Tumor of Bone	125 (100.0)	59 (47.2)	66 (52.8)	0.592	35 (31, 10-79)	48 (32, 13-76)	34 (29, 10-79)	0.642
Plasmacytoma, NOS	154 (100.0)	105 (68.2)	49 (31.8)	<0.001	58 (57, 11-85)	59 (58, 11-85)	55 (55, 20-75)	0.043
Others and Unspecified	1342(100.0)	682 (56.3)	529 (43.7)	0.567	49 (52, 0-98)	46 (47, 0-98)	49 (52, 0-96)	0.728
Total	4112(100.0)	2445(59.5)	1667 40.5)	<0.001	36 (28, 0-98)	36 (27, 0-98)	36 (31, 0-96)	0.915

* The p-value of exact binomial test for comparing of the frequency between males and females by histology subtype with sample size higher than 20.

** The p-value of exact Mann-Whitney U for comparing the mean rank of age between males and females by histology subtypes with sample size higher than 20

The total ASIR (95% CI) was 8.23 (7.98-8.49) per million person-years, while males had the ASIR of 9.67 (95% CI: 9.27-10.06), and females had the ASIR of 6.80 (95% CI: 6.46-7.13) per million person-years. The osteosarcoma, chondrosarcoma, and Ewing sarcoma had the ASIR higher than 1 per million person-years with values of 2.36 (95% CI: 2.23-2.49), 1.26 (95% CI: 1.16-1.37), and 1.08 (95% CI: 0.99-1.17), respectively (table 2). ASIRs of different histological subtypes of bone sarcomas are shown in (table 2). According to the results, long bones of the lower limb were the most affected area, with the ASIR of 3.18 (95% CI: 3.02-3.33) per million person-years. C41.9 was other topography with ASIR higher than 1 (ASIR=2.00; 95% CI: 1.87-2.13) per million person-years. Males were more affected than females in all topography types (table 3). 60.38% of patients were between 10 to 44 years old. Accordingly, 66.38%, 61.86%, 73.28% and 50.7% of tumors in C40.0, C40.1, C40.2, C40.3 were between 10 to 44 years old, respectively. Frequencies and age-specific incidence rates of bone sarcomas in different topographic areas based on age groups are shown in table 4. Long bones of the lower limb were the most commonly affected area in patients younger than 75 years. In those who were 75 years or older, bone, cartilage, joint and articular cartilage of limb were the most commonly affected areas.

**Figure 1:**
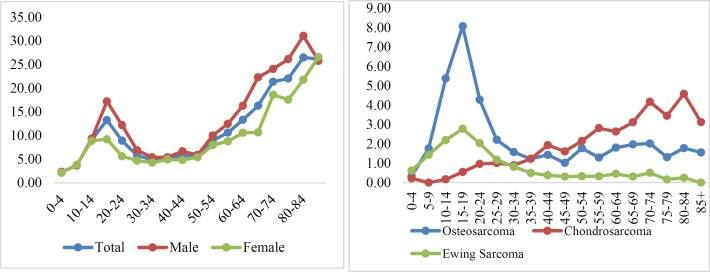
The age specific incidence rate A) of total bone sarcoma during 2008-2014 B) of the most important morphology codes

**Figure 2:**
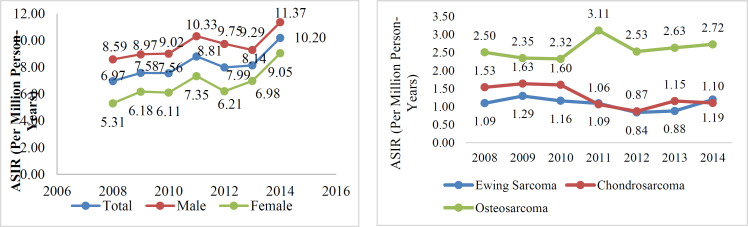
The ASIR (per million person-years) A) of total bone sarcoma during 2008-2014 B) of the most important morphology codes

According to the results, an upward trend of age-specific incidence rate was seen in patients with the age of 5-19 years and those who were older than 55 years (figure 1-A). The highest incidence rates in patients aged 0-14 years were related to osteosarcoma and Ewing sarcoma, however, chondrosarcoma was ranked first for those older than 19 years (figure 1-B). The ASIR trend in bone sarcoma was generally bullish, but because the number of years considered was not enough, we cannot talk about the significance of this trend (figure 2-A). In addition, osteosarcoma generally had a higher incidence rate than other histology subtypes over study years (figure 2-B).

**Table 2:** The ASIR by sex for morphologies

**Morphology Type**	**ASIR per Million Person-Years** ** (95% Confidence Interval)**		
	**Total**	**Male**	**Female**
Osteosarcoma	2.36 (2.23-2.49)	2.82 (2.62-3.02)	1.89 (1.73-2.06)
Chondrosarcoma	1.26 (1.16-1.37)	1.49 (1.34-1.65)	1.03 (0.90-1.16)
Ewing Sarcoma	1.08 (0.99-1.17)	1.29 (1.16-1.43)	0.87 (0.75-0.98)
Giant Cell Tumor of Bone	0.23 (0.19-0.27)	0.22 (0.16-0.28)	0.25 (0.18-0.31)
Plasmacytoma, NOS	0.37 (0.31-0.43)	0.51 (0.41-0.61)	0.23 (0.17-0.30)
Others and Unspecified	2.92 (2.76-3.08)	3.32 (3.08-3.57)	2.53 (2.32-2.74)
Total	8.23 (7.98-8.49)	9.67 (9.27-10.06)	6.80 (6.46-7.13)

**Table 3:** The frequency (percentage) and ASIR based on sex and topographies

**Topography Type**	**Frequency (Percentage)**			**ASIR per Million (95% C.I)**		
	**Total**	**Male**	**Female**	**Total**	**Male**	**Female**
40.0^*^	345	195 (56.5)	150 (43.5)	0.67 (0.60-0.74)	0.74 (0.64-0.85)	0.60 (0.50-0.70)
40.1	97	45 (45.9)	52 (54.1)	0.19 (0.15-0.23)	0.18 (0.12-0.23)	0.21 (0.15-0.26)
40.2^*^	1684	1007 (59.8)	677 (40.2)	3.18 (3.02-3.33)	3.69 (3.46-3.92)	2.65 (2.45-2.86)
40.3^*^	97	63 (64.9)	34 (35.1)	0.20 (0.16-0.24)	0.26 (0.19-0.32)	0.13 (0.09-0.18)
40.8	5	3 (60.0)	2 (40.0)	0.01 (0.00-0.02)	0.02 (0.00-0.03)	0.01 (0.00-0.02)
40.9^*^	319	179 (56.1)	140 (43.9)	0.70 (0.62-0.78)	0.79 (0.67-0.91)	0.62 (0.51-0.72)
41.3^*^	186	121 (65.1)	65 (34.6)	0.38 (0.33-0.44)	0.51 (0.41-0.60)	0.26 (0.20-0.33)
41.4^*^	450	277 (61.6)	173 (38.4)	0.88 (0.80-0.96)	1.09 (0.96-1.22)	0.67 (0.57-0.77)
41.8	10	8 (80.0)	2 (20.0)	0.02 (0.01-0.04)	0.04 (0.01-0.07)	0.01 (0.00-0.02)
41.9^*^	919	547 (59.5)	372 (40.5)	2.00 (1.87-2.13)	2.36 (2.16-2.56)	1.64 (1.47-1.81)
Total^*^	4112 (100.0)	2445 (59.5)	1667 (40.5)	8.23 (7.98-8.49)	9.67 (9.27-10.06)	6.80 (6.46-7.13)

*Significant difference in frequency between male and female at 0.05 level (two tailed) using binomial proportion test

**Table 4:** The frequency (age specific incidence rate per million person-years) of age groups based on topography codes

**Age Group**	**Topography codes (C Codes)**									
	**40.0**	**40.1**	**40.2**	**40.3**	**40.8**	**40.9**	**41.3**	**41.4**	**41.8**	**41.9**
0-4	13(0.30)	3 (0.07)	34 (0.78)	0 (0.00)	0(0.00)	5 (0.11)	0 (0.00)	21(0.48)	1(0.02)	20 (0.46)
5-9	14(0.35)	4 (0.10)	87 (2.16)	5 (0.12)	0(0.00)	7 (0.17)	6 (0.15)	7 (0.17)	0(0.00)	20 (0.50)
10-14	32(0.78)	6 (0.15)	217(5.30)	6 (0.15)	0(0.00)	17(0.42)	11(0.27)	28(0.68)	1(0.02)	55 (1.34)
15-19	54 (1.15)	12 (0.25)	381 (8.08)	14 (0.30)	0 (0.00)	23 (0.49)	15 (0.32)	46 (0.98)	0 (0.00)	81 (1.72)
20-24	46 (0.81)	12 (0.21)	248 (4.35)	13 (0.23)	0 (0.00)	26 (0.46)	22 (0.39)	68 (1.19)	0 (0.00)	73 (1.28)
25-29	26 (0.45)	11 (0.19)	160 (2.74)	6 (0.10)	0 (0.00)	20 (0.34)	17 (0.29)	42 (0.72)	0 (0.00)	56 (0.96)
30-34	25 (0.51)	6 (0.12)	100 (2.05)	6 (0.12)	0 (0.00)	11 (0.23)	6 (0.12)	41 (0.84)	0 (0.00)	41 (0.84)
35-39	19 (0.48)	6 (0.15)	73 (1.83)	5 (0.13)	0 (0.00)	13 (0.33)	14 (0.35)	30 (0.75)	1 (0.03)	45 (1.13)
40-44	27 (0.79)	7 (0.21)	64 (1.88)	5 (0.15)	0 (0.00)	11 (0.32)	12 (0.35)	25 (0.73)	0 (0.00)	45 (1.32)
45-49	14 (0.49)	5 (0.18)	47 (1.65)	10 (0.35)	1 (0.04)	15 (0.53)	13 (0.46)	22 (0.77)	0 (0.00)	34 (1.19)
50-54	19 (0.79)	5 (0.21)	70 (2.90)	6 (0.25)	1 (0.04)	20 (0.83)	16 (0.66)	17 (0.70)	2 (0.08)	61 (2.52)
55-59	16 (0.87)	3 (0.16)	61 (3.30)	4 (0.22)	1 (0.05)	14 (0.76)	17 (0.92)	33 (1.79)	1 (0.05)	46 (2.49)
60-64	11 (0.83)	3 (0.23)	41 (3.09)	2 (0.15)	1 (0.08)	25 (1.88)	11 (0.83)	22 (1.66)	1 (0.08)	60 (4.52)
65-69	10 (1.04)	3 (0.31)	29 (3.01)	5 (0.52)	0 (0.00)	19 (1.97)	11 (1.14)	15 (1.56)	1 (0.10)	64 (6.64)
70-74	11 (1.39)	4 (0.51)	27 (3.41)	7 (0.89)	1 (0.13)	24 (3.04)	8 (1.01)	13 (1.64)	1 (0.13)	73 (9.23)
75-79	5 (0.82)	3 (0.49)	17 (2.80)	1 (0.16)	0 (0.00)	28 (4.61)	4 (0.66)	13 (2.14)	0 (0.00)	63 (10.36)
80-84	1 (0.25)	1 (0.25)	16 (4.08)	0 (0.00)	0 (0.00)	29 (7.39)	2 (0.51)	6 (1.53)	0 (0.00)	49 (12.48)
85+	2 (0.78)	3 (1.17)	12 (4.69)	2 (0.78)	0 (0.00)	12 (4.69)	1 (0.39)	1 (0.39)	1 (0.39)	33 (12.89)

## Discussion

This is the first study in Iran evaluating the epidemiology and incidence rate of bone sarcomas based on age, gender, and tumor location in Iran. Our findings showed that, osteosarcoma, chondrosarcoma and Ewing sarcoma were ranked first to third in terms of ASIR from 2008 to 2015, respectively. In addition, the ASIR was higher in men than women in these histology subtypes. The C40.1 and C40.9 were two common topographies. Two age ranges were more important in this study. The first was the age group of 15-19 years and the second is the age groups after 50 years. Accordingly, bone sarcoma had a higher incidence rate in both ranges. In addition, OS and CS had the highest age-specific incidence rate in the first and second age groups, respectively. In our study, osteosarcoma was the most common bone sarcoma, followed by chondrosarcoma and Ewing sarcoma. This finding is in line with previous studies on sarcomas in Taiwan (19), Japan( 20), and the United Kingdom (UK) (21) as they found similar order of prevalence for bone sarcomas. Solooki et al. evaluated the prevalence of musculoskeletal tumors in Shiraz in the southern part of Iran between 1997 to 2008. In their study, Ewing sarcoma was more prevalent compared to chondrosarcoma (15.9% vs. 8% of all malignant tumors) (11). Different prevalence of bone sarcomas in Solooki et al.’s study compared to our study may be due to bone sarcomas’ different epidemiology in Shiraz compared to Iran. We also evaluated bone sarcomas’ incidence between 2008 to 2015 and Solooki et al. evaluated patients diagnosed with musculoskeletal tumors between 1997 to 2008. There may be a change in the epidemiology of bone sarcomas in Iran. In our study, except in 2014 and 2011, ASIR was higher for chondrosarcoma than for Ewing sarcoma, which may have led to a higher prevalence of chondrosarcoma compared to Ewing sarcoma between 2008 to 2015. With increase in the life expectancy and the fact that in contrast to Ewing sarcoma, chondrosarcoma has higher incidence rate among older adults, prevalence of chondrosarcoma may further increase in the future compared to Ewing sarcoma. 

ASIR for all types of bone sarcomas was 8.23 in our study, which is higher than studies in Taiwan, the UK, and Switzerland with ASIRs of 6.7, 7.5, and 0.91, respectively (16, 21, 22). Such finding indicated a higher incidence of bone sarcomas among the Iranian population, which needs more attention. ASIR of osteosarcoma was 2.36 in our study. Mirabello et al. evaluated osteosarcoma’s global incidence rate and found that global ASIR is 3.4 and 4.3 per million people in females and males, respectively (23). ASIR of osteosarcoma is lower in our study compared to the global average. The lower incidence of osteosarcoma in the Iranian population may be due to genetic factors, as genetic susceptibility is a risk factor for osteosarcoma. ASIR of Ewing sarcoma was 1.08, which is similar to the values reported in the UK (ASIR=1.2) and India (ISIR of 1 to 1.6) (21,24) but is significantly higher compared to Hung study in Taiwan (ASIR= 0.52) (16).

The lower incidence of Ewing sarcoma in Taiwan may be due to ethnicity as Ewing sarcoma’s incidence is relatively low in blacks and east and southeast counties in Asia (9). ASIR of chondrosarcoma in our study was similar to Hung’s study in Taiwan( 16) with ASIR of 1.26 and 1.2, respectively. On the other hand, chondrosarcoma incidence rates were significantly higher in studies conducted in the UK (ASIR=2) and Netherland (incidence rate = 8.78 per million). 

In our study, osteosarcoma and Ewing sarcoma were more common among children and adolescents, but chondrosarcoma, giant cell tumors, and plasmacytoma were mostly seen in older adults in line with previous studies (16,20,25). We did not find the bimodal age distribution for osteosarcoma as there was a peak in the ASIR in patients aged 15 to 19 years, but we did not find any other peak of osteosarcoma incidence. In contrast, Ottaviani et al. found a bimodal age distribution for osteosarcoma with the first peak in patients 10 to 14 years old and the second peak in patients older than 65 (26). 

ASIR for bone sarcomas was higher in males than females among the Iranian population of our study. Gender distribution in our study is similar to other previous studies conducted in other countries as bone sarcomas were seen in males more frequently than female s(16,20,21). Long bones of the lower limb were the most commonly affected area by bone sarcomas, followed by pelvic and upper limbs. In Whelan’s study in the UK, although the lower limb was the most affected area, similar to our study, bone sarcomas’ prevalence in the upper limb was higher than the pelvic (21). Like Whelan’s study, we found an increased incidence of bone sarcomas in pelvic with age. 

We found an increasing trend in the incidence of bone sarcomas in Iran from 8.59 in 2007 to 11.37 per million person-year in 2015. Similarly, the incidence of osteosarcoma and Ewing sarcoma has increased in recent years, in contrast to chondrosarcoma, which its incidence has decreased. Such a trend in the incidence of bone sarcomas is similar to previous studies conducted in other countries such as Japan and Taiwan, as they have found a similar increasing trend in the incidence of bone sarcomas in recent years (16,27). However, the incidence of bone sarcomas has not changed significantly in most countries (28). Future studies are needed to determine the factors affecting bone sarcomas’ incidence in different populations considering the heterogeneity in the bone sarcomas’ incidence trend.

**Limitations**

Our main limitation is that we did not evaluate bone sarcomas’ survival, which is an essential aspect of epidemiological studies. Future studies are indicated to evaluate and compare the survival of different bone sarcomas in Iran and factors affecting survival. Also, we did not have the data regarding the incidence of bone sarcomas after 2015, which may have different epidemiological features. Studies are needed to have evaluated patients’ incidence and epidemiological characteristics with bone sarcomas in recent years. 

This study provided the epidemiological features of bone sarcomas, including the histological type of sarcoma, tumors’ location, and patients’ age and gender in the Iranian population for the first time. We also found an increasing trend in the incidence of bone sarcomas, especially osteosarcoma and Ewing sarcoma, which should be considered for future policies regarding bone sarcomas regarding sarcomas in Iranian people. 
